# The Effect of a Resistance Training Session on Physiological and Thermoregulatory Measures of Sub-maximal Running Performance in the Heat in Heat-Acclimatized Men

**DOI:** 10.1186/s40798-019-0195-y

**Published:** 2019-06-04

**Authors:** Kenji Doma, Anthony Nicholls, Daniel Gahreman, Felipe Damas, Cleiton Augusto Libardi, Wade Sinclair

**Affiliations:** 1 College of Healthcare Sciences, James Cook University, James Cook Drive, Rehab Sciences Building, Townsville, QLD 4811 Australia; 2 Exercise and Sport Science, Charles Darwin University, Casuarina, Australia; 3 MUSCULAB - Laboratory of Neuromuscular Adaptations to Resistance Training, Department of Physical Education, Federal University of São Carlos – UFSCar, São Carlos, Brazil

**Keywords:** Running economy, Creatine kinase, Strength training, Core temperature, Delayed-onset of muscle soreness

## Abstract

**Background:**

The current study examined the acute effects of a lower body resistance training (RT) session on physiological and thermoregulatory measures during a sub-maximal running protocol in the heat in heat-acclimatized men. Ten resistance-untrained men (age 27.4 ± 4.1 years; height 1.78 ± 0.06 m; body mass 76.8 ± 9.9 kg; peak oxygen uptake 48.2 ± 7.0 mL kg^−1^ min^−1^) undertook a high-intensity RT session at six-repetition maximum. Indirect muscle damage markers (i.e., creatine kinase [CK], delayed-onset muscle soreness [DOMS], and countermovement jump [CMJ]) were collected prior to, immediately post and 24 and 48 h after the RT session. The sub-maximal running protocol was performed at 70% of the ventilatory threshold, which was conducted prior to and 24 and 48 h following the RT session to obtain physiological and thermoregulatory measures.

**Results:**

The RT session exhibited significant increases in DOMS (*p* < 0.05; effect size [ES]: 1.41–10.53), whilst reduced CMJ (*p* < 0.05; ES: − 0.79–1.41) for 48 h post-exercise. There were no differences in CK (*p* > 0.05), although increased with moderate to large ES (0.71–1.12) for 48 h post-exercise. The physiological cost of running was increased for up to 48 h post-exercise (*p* < 0.05) with moderate to large ES (0.50–0.84), although no differences were shown in thermoregulatory measures (*p* > 0.05) with small ES (0.33).

**Conclusion:**

These results demonstrate that a RT session impairs sub-maximal running performance for several days post-exercise, although thermoregulatory measures are unperturbed despite elevated muscle damage indicators in heat-acclimatized, resistance untrained men. Accordingly, whilst a RT session may not increase susceptibility to heat-related injuries in heat-acclimatized men during sub-maximal running in the heat, endurance sessions should be undertaken with caution for at least 48 h post-exercise following the initial RT session in resistance untrained men.

## Key Points

- Symptoms of muscle damage have been reported to further increase core temperature during sub-maximal running at a given workload in non-heat acclimatized individuals
- In the current study, exercise-induced muscle damage impaired running economy, although core temperature was unaffected amongst heat-acclimatised individuals
- Although heat-acclimatised individuals may be less susceptible to heat-related illness when running during periods of exercise-induced muscle damage, at least 48 h of recovery is recommended following the initial bout of resistance training prior to undertaking a running session, to optimise training quality

## Background

Exercise-induced muscle damage (EIMD) impairs running economy (RE) measures by elevating oxygen cost of running at sub-maximal intensities [1, 2]. Whilst these findings may have implications for the quality of running session during periods of EIMD [3, 4], the majority of studies examining the impact of EIMD on determinants of running performance have incorporated isokinetic, eccentric contractions [5, 6] or downhill running protocols [1, 7]. This approach does not replicate real-world resistance training (RT) practices involving isoinertial, concentric and eccentric, multi-articular contractions with heavy external loads. More recently, studies have reported that lower body RT sessions, such as squats, leg press, leg extension and leg curls, produced symptoms of EIMD [8], and as a result, impaired RE measures and maximal effort running performance for 24–48 h post-exercise [9–11]. However, these studies examined running performance measures in thermo-neutral conditions. As athletes experience greater thermal strain in hot and humid conditions [12, 13], and the symptoms of EIMD increases physiological cost during exercise at a given workload [11, 14], running during periods of EIMD in the heat may further augment thermal strain.

Indeed, Montain and colleagues [15] showed greater physiological cost of running with increased core temperature (T_C_) measures 7 h after a RT session, although such findings were not reported at 26 h post-exercise. In addition, a correlation was identified between changes in metabolic rate and T_C_ measures during running, indicating that impaired RE may in part have contributed to elevated levels of T_C_. Whilst these findings suggest increased susceptibility of exertional heat-illness during periods of EIMD in resistance-untrained individuals, only two eccentrically biased resistance exercises were performed [15]. This type of training practice does not represent lower body RT sessions consisting of multiple isoinertial, concentric and eccentric exercises [16], which have been shown to impair RE for 24–48 h post-exercise in resistance-untrained men in thermo-neutral conditions [11]. Fortes et al. [17] reported increased physiological cost of running with elevated T_C_ in resistance-untrained individuals for up to 24 h during periods of EIMD. However, the symptoms of EIMD were caused using downhill running, and participants were non-heat acclimatized. Given that thermoregulation is highly dependent on previous exposure to conditions in the heat [18], the thermal responses of exercise may differ in heat-acclimatized individuals.

Subsequently, the purpose of the current study was to examine the acute effect of a lower body RT session on thermal responses and RE measures during sub-maximal running, in heat-acclimatized individuals. We hypothesized that an isoinertial, lower body RT session would augment indirect muscle damage markers, and as a result, increase thermal strain during sub-maximal running. These findings may shed light on the recovery dynamics between resistance and endurance training sessions during concurrent training (i.e. combination of resistance and endurance bouts in the one training program) in the heat [19], and assist practitioners to optimize training prescription and minimize the susceptibility to heat illness in endurance athletes.

## Methods

### Participants

Ten healthy men (mean ± standard deviation; age 27.4 ± 4.1 years; height 1.78 ± 0.06 m; body mass 76.8 ± 9.9 kg; maximal oxygen uptake [VO_2max_] 48.2 ± 7.0 mL kg^−1^ min^−1^) volunteered for this study. According to an a priori calculation, ten participants were sufficient to generate a power of 0.8 at an alpha level of 0.05 for the dependent measures based on previous studies [2, 11, 17]. Each participant had lived in a tropical climate of North Queensland, Australia, for at least 3 years and were undertaking regular (2–3 week^−1^) running sessions outside, but had not performed lower body RT for the past 6 months. Biological variations were controlled for by refraining from high-intensity activity for at least 48 h prior to any testing session; avoiding caffeine and food intake for at least 2 h prior to any testing session; wearing the same shoes for every testing session; conducting each training and testing session at the same time of day within participants; and refraining from recovery-related activities, such as supplementation, medication, massage and cryotherapy, during the course of the study. Each participant provided written informed consent prior to taking part in any testing procedures, and did not report acute or chronic illness, disease and injury or medication that would contraindicate any training and testing procedures. The Institutional Human Research Ethics Committee approved all protocols, which were in line with the Declaration of Helsinki.

### Research Design

**Fig. 1:**
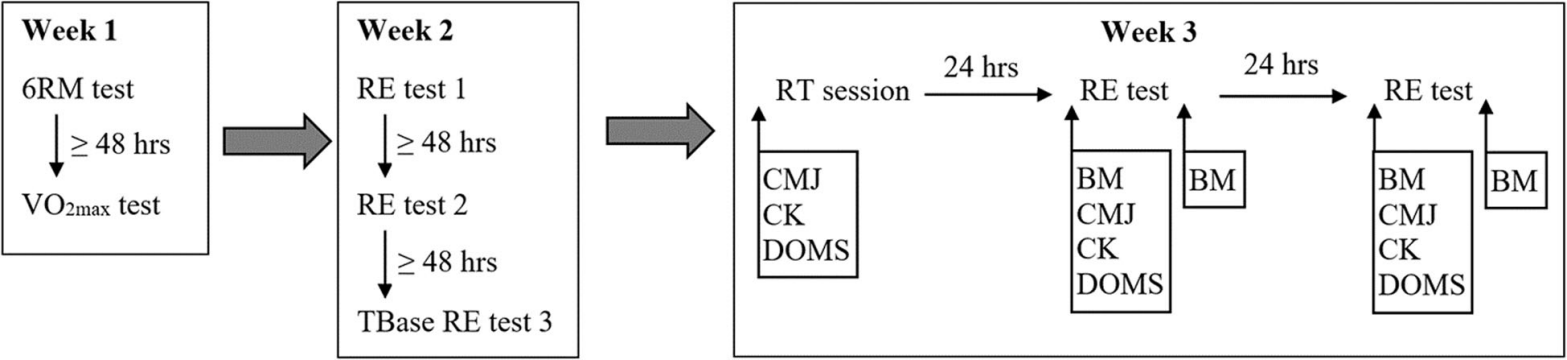
The schematic of the research design including the 6 repetition maximum (6RM) test, maximal oxygen consumption (VO2max) test, running economy (RE) test at baseline (TBase), resistance training (RT), countermovement jump (CMJ), creatine kinase (CK), delayed onset of muscle soreness (DOMS) and body mass (BM)

This study was conducted as a repeated measures design across 3 weeks (Fig. 1). The first week consisted of a familiarization session, followed by a VO_2max_ test 48 h thereafter. The familiarization session ensured each participant was acquainted with the procedures and equipment as well as to undertake a six-repetition maximum (6RM) test. During the second week, three RE tests, with at least 48 h of recovery in-between each testing sessions, were conducted to ensure familiarity with the RE protocol. The third RE test during the second week was used to report on baseline measures TBase. During the third week, each participant undertook a RT session. Subsequent RE tests were performed 24 (T_24_) and 48 (T_48_) hours following the RT session, which were then compared to TBase. Indirect muscle damage markers were also collected prior to (TBase), immediately post (T1) and 24 (T24) and 48 (T48) hours following the RT bout.

### Six-Repetition Maximum Assessment

The 6RM protocol was conducted using exercises in the order of squats on a Smith Machine (MPL 706, Maxim Fitness, Australia), horizontal leg press (NS4000, Nautilus, Canada), leg extension (NS4000, Nautilus, Canada) and leg curls (NS4000, Nautilus, Canada) in the same session. Prior to the 6RM assessment, each participant undertook a standardized warm-up by completing ten repetitions of leg swings in the frontal and sagittal planes for each leg, followed by ten repetitions of squat exercises on the Smith Machine at approximately 50% of body mass. The 6RM protocol was conducted using previously described methods [20]. In summary, each participant completed 8–10 repetitions with a load approximately at 10RM based on perception of effort during the warm-up. Following 5 min of passive rest, the load was increased by 20% to attempt a 6RM. The load was adjusted by 5–10% heavier, or lighter, depending on the participant’s perceived load. A 5-min passive recovery period was provided between each attempt. The squats and horizontal leg press commenced with the knees fully extended at the start position and flexed to 45° at the end of the eccentric phase. Each participant was also requested to complete each repetition in approximately 2 s with 1 s for concentric and eccentric phases, respectively, to standardize contraction speed. In addition, the leg press exercise was performed unilaterally, commencing with the right leg. A qualified strength and conditioning coach ensured proper technique and correct loading was applied during each testing session. The highest load attempt in the 6RM test was considered for the calculations of training load of the RT protocol described below.

### VO_2max_ Test

The VO_2max_ test was conducted in a custom-built climate-controlled chamber with the temperature and humidity set at 30 °C and 67.5%, respectively. These temperature and humidity settings were selected to replicate the average tropical environmental constraints of Far North Queensland, Australia. Prior to the VO_2max_ test, a progressive warm-up was conducted. The warm-up activities included dynamic stretches of the lower extremity; jogging at 8, 10 and 12 km h^−1^ for 1 min, respectively; 2 min of walking at 5 km h^−1^ on a treadmill (TM 601, Trackmaster, USA); and 1 min of passive recovery. The VO_2max_ test was conducted using a continuous incremental protocol [21], commencing at 9 km h^−1^ and was increased by 1.5 km h^−1^ every minute until volitional exhaustion was reached using verbal encouragement. The participants were deemed to have been reached VO_2max_ if there was no increase in VO_2_ (mL kg min^−1^), despite an increase in treadmill speed, or if the following criteria was achieved: respiratory exchange ratio > 1.1, maximum heart rate (HR) within ten beats of the age-appropriate reference value and Borg’s rating of perceived exertion of > 18 [22]. During the VO_2max_ test, an indirect calorimetry system (Quark CPET, Cosmed, Italy) was used to collect expired air and calculate the second ventilatory threshold (VT_2_). The VT_2_ was quantified from the inflection point of ventilation (VE) with respect to carbon dioxide production (VCO_2_) on a scatter diagram from the VO_2max_ test [23]. The running intensity at VT_2_ was then utilized to establish running speeds during the RE tests.

### Running Economy Test

Similar to the VO_2max_ test, the RE test was conducted in a climate-controlled chamber with identical temperature and humidity settings. Prior to the RE test, a urine sample was collected to determine urine specific gravity using a calibrated urinary refractometer (Atago hand refractometer, model UNC-NE; Atago, Japan). Whole body sweating rate was also measured prior to, and following, each RE test to report changes as a result of the RE test. Due to technical difficulties with data collection, body mass change was reported for nine participants. Following an identical warm-up to the VO_2max_ test, the RE test was completed at 70% of VT_2_ for 10 min [24]. During the RE protocol, respiratory measures were collected using an indirect calorimetry system (Quark CPET, Cosmed, Italy) to report oxygen consumption (VO_2_; mL kg^−1^ min^−1^), carbon dioxide (VCO_2_; mL kg^− 1^ min^−1^), respiratory exchange ratio (RER), breath frequency (Bf; breath·min^−1^) and ventilatory equivalents for oxygen (VE/VO_2_) and carbon dioxide (VE/VCO_2_). These respiratory measures were reported as the average of the last 3 min of the RE test. Potential existence of a VO_2_ slow component was also assessed for the baseline by comparing VO_2_ between the 7th and 10th minute of the RE test. The primary RE parameter was based on the gross caloric unit cost (CU_C_) of running, using a previously reported method [25]. Initially, the caloric equivalent of VO_2_ was determined, and the CU_C_ was then calculated as caloric unit cost (Kcal kg^−1^ min^−1^) = VO_2_·caloric equivalent·s^−1^·BM^−1^·K, where VO_2_ was in litres per minute, caloric equivalent was in kilocalories per litre, speed (s) was in metres per minute, body mass (BM) in kgs and K in 1000 m km^−1^. Core temperature (T_C_) was obtained by having participants ingest a telemetry pill (CorTemp; HQInc, Palmetto, USA) 8 h prior to each test. Heart rate (HR; RS800CX, Kempele, Finland), 6–20 Borg’s rating of perceived exertion (RPE), thermal discomfort (D_T_) and thermal sensation (S_T_) were also recorded on the 9th minute. The D_T_ was measured using a 1–5 visual analogue scale with 1 and 5 denoting ‘comfortable’ and ‘extremely uncomfortable’, respectively. The S_T_ was also a visual analogue scale, ranging from 1 to 13, with 1 and 13 denoting ‘unbearably cold’ and ‘unbearably hot’, respectively. All measures obtained from the RE test were reported from baseline to 48 h post-exercise. However, T_C_ will be reported for 24 h post-exercise due to participants voiding the sensor prior to the 48 h collection point.

### Resistance Training Session

The resistance exercises undertaken during the RT session were equivalent, and in the same order as the 6RM session. For each exercise, three sets of six repetitions were performed at 95% of 6RM to ensure that participants were able to complete each set without failure. Two minutes of passive recovery was provided in-between each set and exercise. During the RT session, participants rated the level of difficulty of each set from 1 to 10, with 1 and 10 denoting ‘very easy’ and ‘very difficult’, respectively [2]. Whilst no participants rated their level of difficulty below 8 following the second set, if participants rated their difficulty below 9 during the second set, the load was increased by 5% to ensure adequate stress. As a result, no participants rated their difficulty below 9 following the final set of each exercise.

### Indirect Markers of Muscle Damage

The indirect muscle damage markers were countermovement jump (CMJ), creatine kinase (CK) and delayed onset of muscle soreness (DOMS). For the CMJ, participants undertook three jumps that were measured using a vertical jump apparatus (Yard Stick, Swift Performance, Australia). Participants were given at least 30 s of passive rest in-between each attempt, with the best score reported. Excellent test-retest reliability (intra-class correlation coefficient of 0.92) has previously been reported with an identical CMJ protocol for a similar group of moderately endurance-trained men [2, 20]. For CK, a 30 μL blood sample was collected via finger prick following 10 min of supine rest in a thermo-neutral condition of 22–23 °C. The blood sample was pipetted to a test strip and assessed for CK using a colorimetric assay method (Reflotron, Boehringer Mannheim, Germany). An in-house intra-assay coefficient of variation for CK within our laboratory was 7.2%. The DOMS was obtained using a 1–10 visual analogue scale with 1 denoted as ‘no soreness’ and 10 as ‘very, very sore’ [20]. To standardize the context of DOMS assessment, participants were requested to perform one repetition of a body weight squat and report the number of their perceived DOMS in the scale.

### Statistical Analyses

The measure of central tendency and dispersion for all data are reported as means ± standard deviation using Statistical Package of Social Sciences (SPSS, version 25; IBM Corp., Armonk, USA) software. The Shapiro-Wilk test was used to examine normality of the data distribution, and only CK and DOMS measures were departed from the norm. Thus, a one-way repeated measures analysis of variance (ANOVA) was employed for the majority of measures, with Bonferroni’s pairwise comparisons to determine the location of differences between each time point (i.e. TBase, T1 (only the indirect muscle damage markers), T24 and T48). A Friedman test was used to compare DOMS and CK across time points, with a Man-Whitney *U* test when a main time effect was identified. A paired sample *t* test was conducted for T_C_, given that measures were only collected at TBase and T24 and for VO_2_ slow component between the 7th and 10th minute of the RE test. Effect size (ES, Cohen’s *d*) was also calculated to report on the magnitude of differences between each time point, with 0.2, 0.5 and 0.8 classified as small, moderate and large, respectively [26]. The alpha level was set at 0.05 for all analyses.

## Results

The changes in body mass were significantly greater at T24 (− 1.24 ± 0.38 kg; *p* = 0.03; ES = 0.60) and T48 (− 1.34 ± 0.36 kg; *p* = 0.01; ES = 0.76) when compared to TBase (− 1.08 ± 0.30 kg). However, there were no differences between TBase (1.007 ± 0.007 g mL^−1^), T24 (1.009 ± 0.010 g mL^−1^) and T48 (1.010 ± 0.008 g mL^−1^) for hydration status (*p* = 0.14; *F* = 2.19). Furthermore, no differences were identified (*p* = 0.46) in VO_2_ between the 7th (35.4 ± 3.5 mL kg min^−1^) and 10th (35.3 ± 2.6 mL kg min^−1^) minute of the RE test.

The RT session induced statistically significant increases in VE (*p* = 0.01), VE/VO_2_ (*p* = 0.03) and VO_2_ (*p* = 0.04) at T24 and in VE/VCO_2_ at T24 (*p* = 0.03) and T48 (*p* = 0.02) with moderate ES (0.51–0.68) during the RE test (Table 1). However, no changes were observed in the remaining RE measures (*p* > 0.05) with small ES (0.02–0.49; Table 2). Similarly, no changes were found in the thermal measures of T_C_ at T24 (*p* = 0.552), D_T_ or S_T_ at T24 (*p* = 0.53 and *p* = 1.00, respectively) and T48 (*p* = 0.08 and *p* = 0.50, respectively) with small ES (0.11–0.46; Table 2). However, HR was greater at T24 with a moderate ES (0.51; Table 2).

**Table 1:** The mean ± standard deviation of the psycho-physiological measures during the running economy test at baseline (TBase), 24 h post (T24) and 48 h post (T48) resistance training bout

	TBase	T24	T48	Time effect
CU_C_ (Kcal kg^−1^ km^−1^)	1.13 ± 0.10	1.19 ± 0.12*	1.16 ± 0.12	*p* = 0.01
R_f_ (min^−1^)	36.8 ± 9.2	39.8 ± 9.0	40.1 ± 8.7	*p* = 0.03
VE (L min^−1^)	73.7 ± 10.8	81.6 ± 11.3*	78.8 ± 9.5	*p* = 0.001
VE/VO_2_	27.7 ± 3.2	29.1 ± 3.2*	28.8 ± 3.3	*p* = 0.01
VE/VCO_2_	28.3 ± 2.9	29.7 ± 3.2*	29.9 ± 3.1*	*p* = 0.004
VO_2_ (mL kg^−1^ min^−1^)	34.9 ± 3.2	36.5 ± 3.5*	35.8 ± 3.0	*p* = 0.007
VCO_2_ (mL kg^−1^ min^−1^)	34.0 ± 3.3	35.9 ± 3.7*	34.4 ± 3.5	*p* = 0.02
RER	0.98 ± 0.03	0.98 ± 0.05	0.97 ± 0.05	*p* = 0.34
RPE	12 ± 2	13 ± 3	13 ± 3	*p* = 0.493
HR (beats·min^−1^)	166 ± 10	171 ± 12	169 ± 10	*p* = 0.073
T_C_ (°C)	37.8 ± 0.3	37.7 ± 0.3	–	–
D_T_	2.55 ± 0.72	2.80 ± 0.82	2.90 ± 0.81	*p* = 0.08
S_T_	9.50 ± 0.85	9.60 ± 1.00	9.80 ± 0.92	*p* = 0.322

*CU*_*C*_ caloric unit cost, *R*_*f*_ respiratory frequency, *VE* ventilation, *VCO*_*2*_ carbon dioxide production, *VE*/*VO*_*2*_ ventilatory equivalents for oxygen, *VE*/*VCO*_*2*_ ventilatory equivalents for carbon dioxide production, *RER* respiratory exchange ratio, *RPE* rating of perceived exertion, *HR* heart rate, *D*_*T*_ thermal discomfort, *S*_*T*_ thermal sensation, *T*_*C*_ core temperature

* Significantly greater than TBase (*p* < 0.05)

**Table 2:** The effect size calculations with associated 95% confidence interval of the psycho-physiological measures during the running economy test between baseline (Tbase) and 24 h (T24) and 48 h (T48) following the resistance-training bout

	TBase-T24	TBase-T48	T24-T48
CU_C_	0.49 (− 0.42–1.36)	0.22 (− 0.67–1.09)	0.25 (− 0.64–1.12)
R_f_	0.34 (− 0.56–1.21)	0.33 (− 0.57–1.19)	0.02 (− 0.85–0.90)
VE	0.68^a^ (− 0.25–1.55)	0.49 (− 0.42–1.36)	0.24 (− 0.65–1.11)
VCO_2_	0.43 (− 0.48–1.30)	0.11 (− 0.77–0.98)	0.33 (− 0.57–1.20)
VE/VO_2_	0.44 (− 0.47–1.31)	0.34 (− 0.56–1.21)	0.09 (− 0.79–0.97)
VE/VCO_2_	0.46 (− 0.45–1.33)	0.53^a^ (− 0.38–1.40)	− 0.06 (− 0.94–0.82)
VO_2_	0.07 (− 0.81–0.94)	0.28 (− 0.61–1.15)	0.03 (− 0.85–0.90)
RER	0.00 (− 0.88–0.88)	− 0.49 (− 1.35–0.42)	0.40 (− 0.50–1.27)
RPE	0.32 (− 0.57–1.19)	0.19 (− 0.70–1.06)	0.12 (− 0.77–0.99)
HR	0.51^a^ (− 0.40–1.38)	0.30 (− 0.59–1.17)	0.24 (− 0.65–1.11)
T_C_	− 0.33 (− 1.20–0.56)	–	–
D_T_	0.31 (− 0.57–1.19)	0.46 (− 0.45–1.32)	− 0.12 (− 0.99–0.76)
S_T_	0.11 (− 0.77–0.98)	0.34 (− 0.56–1.21)	− 0.21 (− 1.08–0.68)

*R*_*f*_ respiratory frequency, *VE* ventilation, *VCO*_*2*_ carbon dioxide production, *VE*/*VO*_*2*_ ventilatory equivalents for oxygen, *VE*/*VCO*_*2*_ ventilatory equivalents for carbon dioxide production, *RER* respiratory exchange ratio, *RPE* rating of perceived exertion, *HR* heart rate, *D*_*T*_ thermal discomfort, *S*_*T*_ thermal sensation, *T*_*C*_ core temperature

^a^Moderate effect

**Fig. 2:**
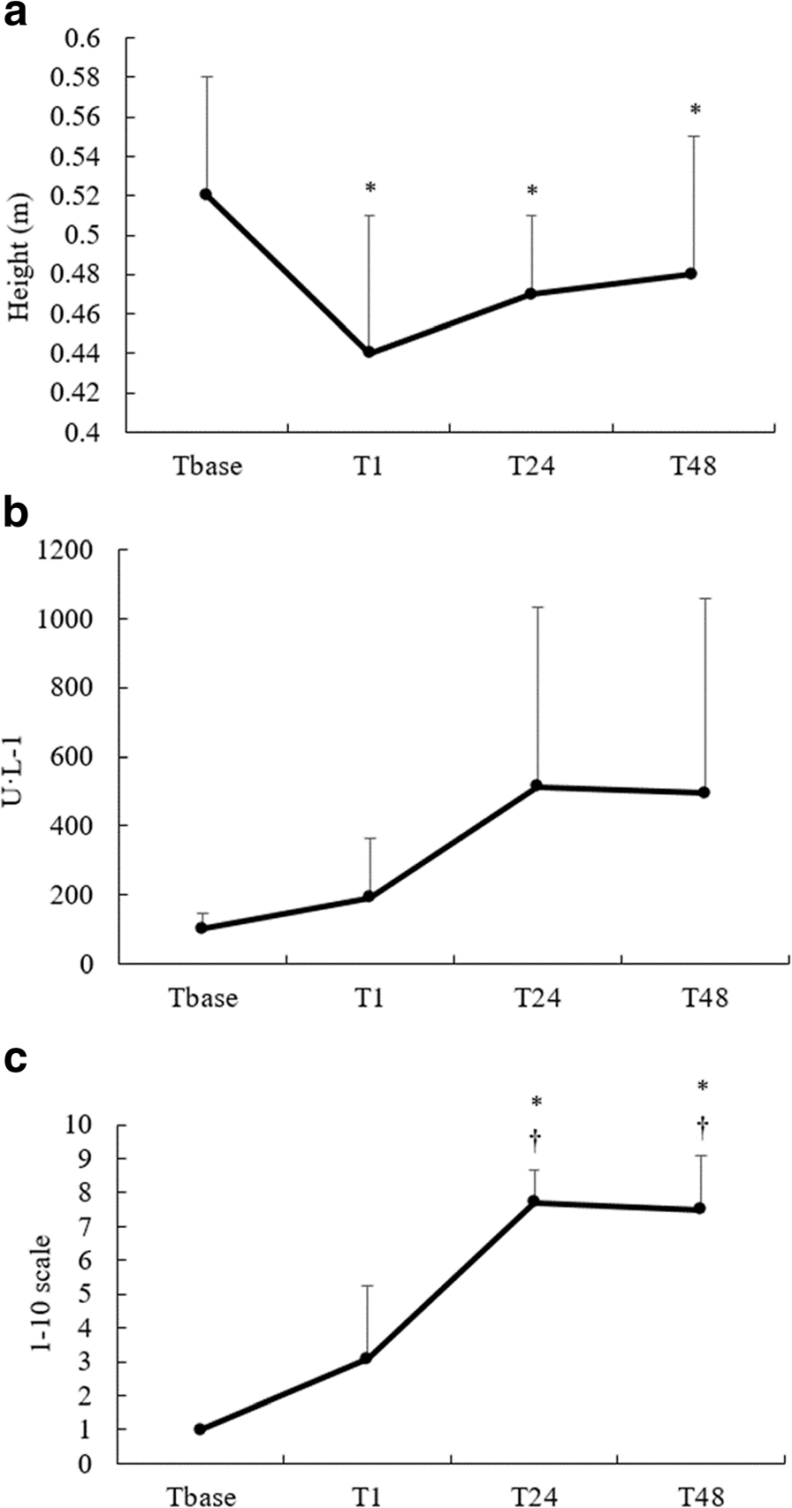
Mean ± standard deviation of the indirect muscle damage markers at baseline (Tbase), immediately following (T1) and 24 (T24) and 48 (T48) hours following the resistance training bout for countermovement jump (**a**), creatine kinase (**b**) and muscle soreness (**c**) measures. * Significantly different from Tbase (*p* < 0.05); † significantly different from T1 (*p* < 0.05)

For the indirect muscle damage markers, there were statistically significant reductions in CMJ at T1, T24 and T48 (*p* < 0.05; Fig. 2) with moderate to large ES (− 0.79–1.41; Table 3), although DOMS was statistically significantly elevated at T24 and T48 (*p* < 0.05), with large ES (1.41–10.53). Whilst no statistically significant differences in CK measures (*p* > 0.05) were identified, differences were exhibited with moderate to large ES (0.71–1.12) for the majority of post-baseline time points.

**Table 3:** The effect size calculations of the indirect markers of muscle damage between baseline (TBase) and immediately post (T1), 24 h (T24) and 48 h (T48) following the resistance-training bout with 95% confidence intervals in parentheses

	CMJ	CK	DOMS
TBase–T1	− 1.41^b^ (− 0.38–2.32)	0.71^a^ (− 0.22–1.58)	1.41^b^ (0.38–2.33)
TBase–T24	− 1.08^b^ (− 0.10–1.96)	1.12^b^ (0.14–2.01)	10.53^b^ (6.84–13.33)
TBase–T48	− 0.79^a^ (− 0.15–1.66)	0.99^b^ (0.02–1.87)	5.75^b^ (3.58–7.42)
T1–T24	0.60^a^ (− 0.32–1.47)	0.84^b^ (− 0.11–1.71)	2.85^b^ (1.51–3.94)
T1–T48	0.65^a^ (− 0.27–1.52)	0.73^a^ (− 0.20–1.60)	2.36^b^ (1.14–3.38)
T24–T48	0.15 (− 0.73–1.02)	− 0.03 (− 0.91–0.85)	− 0.15 (− 1.03–0.73)

*CMJ* countermovement jump, *CK* creatine kinase, *DOMS* delayed onset of muscle soreness

^a^Moderate effect

^b^Large effect

## Discussion

The current study examined the acute effects of a RT session on physiological and thermal measures during sub-maximal running in heat-acclimatized men. The results showed that the RT session resulted in EIMD symptoms whilst concomitantly impaired the physiological cost of running for 24–48 h post-exercise. However, T_C_ and perceptual measures of heat stress remained unchanged during periods of EIMD. These findings suggest that, whilst the quality of running sessions may be compromised with symptoms of EIMD in hot and humid conditions, heat-acclimatized individuals may be less susceptible to exercise-induced heat stress than previously reported for non-heat-acclimatized individuals during sub-maximal running.

One of our primary objectives was to determine whether EIMD caused by resistance exercises altered thermoregulatory responses during sub-maximal running. Periods of EIMD are typically accompanied by increased local inflammatory markers, such as leukocytes, interleukin-1β and interleukin-6 [27]. These immunological responses are believed to alter thermoregulation and augment the risk of exertional heat illness, by increased heat storage and reduced sweating responses [15]. In contrast, the current study showed no changes in thermoregulatory measures, including T_C_, DT and S_T_, during the RE test 24 h following the RT session. These findings also support results of Montain et al. [15], with no changes in T_C_ for 26 h after eccentric-biased lower body resistance exercises in heat-acclimatized men. Conversely, Fortes and colleagues [17] reported higher T_C_ responses 24 h after muscle-damaging exercises (i.e. downhill running), with increased indirect muscle damage makers (i.e. CK and DOMS), in non-heat-acclimatized men.

The similarity in findings between Montain et al. [15] and the current study in heat-acclimatized men, with conflicting results by Fortes et al. [17] in non-heat-acclimatized men, demonstrates that previous exposure to heat-related stress may have an important role in modulating thermoregulation during periods of EIMD. Specifically, heat-acclimatized individuals may have a greater adaptability to thermal stress in response to a heightened physiological cost of running, including VO_2_, VE/VO_2_ and HR. This notion is partly supported by results in the current study with increased body mass loss from the RE test during periods of EIMD, possibly to release more body heat by augmenting sweat rate [28]. It is widely understood that altered sweat response is a principle adaptive response to heat acclimatization during exercise [18], by enhancing skin blood flow to maintain T_C_ within physiologically beneficial levels [29]. In addition, HR response showed moderate ES at 24 h vs TBase (ES = 0.51) in the current study. These findings are greater than Doma et al. [2], who reported only small ES changes (0.35) in HR during the same RE test under thermo-neutral conditions of 22–24 °C, 24–48 h following identical RT protocols. Thus, it is possible that the participants in the current study experienced greater redistribution of blood to the peripheral vascular system at 24 h following the RT session, and as a result, increased HR to compensate for loss in stroke volume to maintain cardiac output [30]. However, more research is needed to confirm whether changes in RE measures are affected by varying ambient environmental conditions during periods of EIMD.

Whilst previous heat-related exposure may have contributed to discrepancies in previous findings on thermoregulatory measures during sub-maximal running, it is important to note that distinct testing protocols were also utilized. For example, Fortes et al. [17] employed downhill running as a muscle-damaging protocol, which is highly eccentric-focused and replicates movement patterns more closely to running than RT. Subsequently, participants in the study by Fortes et al. [17] may have experienced greater mechanical damage in muscle groups essential for running performance. In addition, Fortes et al. [17] utilized a sub-maximal running protocol for 40 min, as opposed to the ~ 10 min of sub-maximal running in the current study. Thus, a longer exposure to hot and humid conditions may have predisposed participants to sustain higher levels of T_C_ in the study by Fortes et al. [17]. Indeed, T_C_ measures continually rise during exercise, with longer periods of exposure in the heat [31].

For indirect EIMD markers, the current study identified a significant reduction in CMJ and elevation in CK and DOMS measures with large ES. Similarly, the respiratory measures exhibited significant increases during the RE test with moderate to large ES, demonstrating that the RT session was sufficient to induce EIMD symptoms and acutely impair determinants of sub-maximal running performance in heat-acclimatized, endurance-trained but resistance-untrained men. Directly comparing these findings to previous studies is at present difficult, given that the current study is the first to examine the acute effect of a resistance exercise bout on determinants of running performance in hot and humid conditions for this population, as far as the authors are aware. However, others have conducted similar studies in thermo-neutral conditions for resistance-untrained men. For example, Doma et al. [2] showed significant increases in indirect EIMD markers of jump height, CK and DOMS measures with a concomitant elevation in VO_2_, VE/VO_2_, VE/VCO_2_, HR and RPE for 24–48 h after a RT session similar to the current study, consisting of squats, single-leg leg press, leg extension and leg curls at 6RM. However, running performance measures were reported only at 90% of anaerobic threshold (AT). Interestingly, Doma et al. [9] reported a significant increase in the physiological cost of running at 70% of AT during a RE test in thermo-neutral conditions 24 h following a bout of lower body resistance exercises, including incline leg press, leg extension and leg curls. It should also be noted that a high-intensity running session was also incorporated 6 h following the RT session on the same day, which appeared to have contributed to changes in RE measures. Collectively, the results of the current and previous studies suggest that a session of lower body RT impairs determinants of sub-maximal running performance amongst resistance-untrained individuals in varying thermal constraints. Therefore, sub-maximal running sessions performed the day after a RT session may impair the quality of training for resistance-untrained individuals, and should be taken with caution at the commencement of a concurrent training program [32].

The mechanisms causing perturbations in RE measures during periods of EIMD are still not fully understood, but it has been speculated that unfamiliar resistance exercise may alter ventilatory response during sub-maximal activity due to symptoms of DOMS [33]. In fact, nocioceptive muscle afferents are activated during DOMS [34], and has been suspected to cause an increase in ventilation during exercise with EIMD [35]. The present results and others [2, 20, 36] support this theory, with augmented measures of R_f_ during RE with EIMD. Thus, DOMS appears to be a sensitive measure during periods of EIMD, and may be a useful monitoring tool for endurance athletes following resistance training sessions.

Whilst the current findings may improve training recommendations for moderately endurance-trained individuals commencing a resistance-training program, a number of limitations should be identified. Firstly, the current study did not incorporate a control condition without a resistance training session, and thus it could be speculated that changes in respiratory measures at T48 may be due to physical stress caused by the RE test the previous day at T24. However, our previous work has demonstrated that RE tests conducted across multiple days consecutively do not alter respiratory measures and RPE in moderately endurance-trained individuals, even at intensities above ventilatory threshold (Doma et al. 2015). Nonetheless, further research would verify whether RE tests conducted in hot and humid conditions compound the effects of EIMD on determinants of running performance. Secondly, the current study employed a resistance training protocol with a moderately heavy load, and the results may not be reproduced following resistance training protocols with heavier or lighter loading methods. Therefore, more research is necessary to determine whether RE measures during periods of EIMD in hot and humid conditions are perturbed by employing other modes of resistance training protocols. Third, the current study solely focused on the impact of EIMD on RE in hot and humid conditions. Future work comparing current findings to a thermal-neutral condition may provide further implications of the impact of EIMD on determinants of running performance in hot and humid conditions in heat-acclimatized individuals. Finally, the core temperature was collected using core temperature pills, which is indicative of gastrointestinal temperature. Whilst temporal changes gastrointestinal temperature are similar via rectal and oesophageal methods [37], further research is necessary to confirm our current findings with other core temperature measures.

## Conclusions

In conclusion, the current study demonstrated that a RT session, consisting of multiple exercises performed with both concentric and eccentric isoinertial phases, impaired RE measures for 24–48 h post-exercise, with no changes in thermoregulatory measures. From a practical standpoint, heat-acclimatized individuals may have less susceptibility to exercise-induced heat stress during periods of EIMD. However, running sessions at sub-maximal intensities should be undertaken with caution in the heat for at least 48 h following a bout of lower body, moderate-to-high intensity (i.e. 6RM) resistance training in endurance-trained, resistance-untrained men to optimize training quality.

## Data Availability

The datasets used and/or analysed during the current study are available from the corresponding author on reasonable request.

## Abbreviations

AT: Anaerobic threshold
Bf: Breath frequency
CK: Creatine kinase
CMJ: Countermovement jump
DOMS: Delayed onset of muscle soreness
D_T_: Thermal discomfort
EIMD: Exercise-induced muscle damage
ES: Effect size
HR: Heart rate
RE: Running economy
RER: Respiratory exchange ratio
RM: Repetition maximum
RPE: Rating of perceived exertion
RT: Resistance training
S_T_: Thermal sensation
T1: Immediately post
T_24_: 24 h post-exercise
T_48_: 48 h post-exercise
TBase: Baseline time point
T_C_: Core temperature
VCO_2_: Carbon dioxide production
VE: Ventilation
VE/VCO_2_: Ventilatory equivalents for carbon dioxide
VE/VO_2_: Ventilatory equivalents for oxygen
VO_2_: Oxygen consumption
VO_2max_: Maximal oxygen uptake
VT_2_: Second ventilator threshold
